# Listening to Black Pregnant and Postpartum People: Using Technology to Enhance Equity in Screening and Treatment of Perinatal Mental Health and Substance Use Disorders

**DOI:** 10.1007/s40615-024-01989-z

**Published:** 2024-04-11

**Authors:** Sara M. Witcraft, Emily Johnson, Anna E. Eitel, Angela D. Moreland, Courtney King, Mishka Terplan, Constance Guille

**Affiliations:** 1 Medical University of South Carolina, Department of Psychiatry and Behavioral Sciences, 67 President Street, MSC 861, 29425 Charleston, SC USA; 2 College of Nursing, Medical University of South Carolina, 99 Jonathan Lucas Street, MSC 160, 29425 Charleston, SC USA; 3 College of Medicine, Medical University of South Carolina, 96 Jonathan Lucas Street, MSC 617, 29425 Charleston, SC USA; 4 Friends Research Institute, 1040 Park Avenue, Ste. 103, 21201 Baltimore, MD USA; 5 Department of Obstetrics and Gynecology, Medical University of South Carolina, Charleston South Carolina, 171 Ashley Ave, 29425 Charleston, SC USA

**Keywords:** Maternal health, Perinatal mental health, Perinatal substance use, Racial disparities, Technology, Qualitative

## Abstract

Perinatal mood and anxiety disorders (PMADs), perinatal substance use disorders (PSUDs), and intimate partner violence (IPV) are leading causes of pregnancy-related deaths in the United States. Screening and referral for PMADs, PSUDs and IPV is recommended, however, racial disparities are prominent: Black pregnant and postpartum people (PPP) are less likely to be screened and attend treatment compared to White PPP. We conducted qualitative interviews to better understand the experience of Black PPP who used a text/phone-based screening and referral program for PMADs/PSUDs and IPV—*Listening to Women and Pregnant and Postpartum People (LTWP)*. We previously demonstrated that LTWP led to a significant reduction in racial disparities compared to in-person screening and referral, and through the current study, sought to identify facilitators of PMAD/PSUD symptom endorsement and treatment attendance. Semi-structured interviews were conducted with 68 Black PPP who were or had been pregnant within the last 24 months, and who either had or did not have a PMAD or PSUD. Participants were enrolled in LTWP and provided feedback on their experience. Using a grounded theory approach, four themes emerged: usability, comfort, necessity, and recommendations. Ease of use, brevity, convenience, and comfort in discussing mental health and substance use via text were highlighted. Need for a program like LTWP in Black communities was discussed, given the reduction in perceived judgement and access to trusted information and resources for PMADs/PSUDs, which may lessen stigma. These qualitative findings illuminate how technology-based adaptations to behavioral health screening and referral can reduce perceived negative judgment and facilitate identification and referral to treatment, thereby more adequately meeting needs of Black PPP.

## Introduction

Maternal mortality, defined as death during pregnancy or the first year postpartum, is higher in the United States (U.S) than all other developed countries [1] and has risen from 20.1 to 23.8 deaths per 100,000 live births between 2019 and 2020 [2]. Although maternal mortality is increasing across all racial/ethnic groups, there are significant and persistent racial disparities [3]. Black pregnant and postpartum people (PPP) experience among the highest pregnancy-related death rate of any racial group, at nearly 4 times that of their White counterparts [2]. The mean maternal mortality rate for Black PPP increased from 26.7 to 55.4 deaths per 100,000 live births between 1999 and 2019, a rate double that of the White PPP mean mortality rate [4].

Mental health conditions are the leading cause of maternal mortality in the U.S. [5]. Recent data from the Centers for Disease Control and Prevention (CDC) across 36 U.S. states found that approximately one-fourth of pregnancy-related deaths are due to suicide and drug overdose [5]. Undetected and untreated Perinatal Mood and Anxiety Disorders (PMADs) and Perinatal Substance Use Disorders (PSUDs) significantly increase the risk of death due to suicide and drug overdose. Cases of maternal deaths due to mental health conditions are commonly characterized by a history of depression (72%), anxiety disorder (48%), and past or current substance use (67%) [6]. In addition to maternal death, PMADs and PSUDs are associated with a number of adverse pregnancy, birth, neonatal, and child outcomes [7–9].

While maternal deaths due to mental health conditions are more common among White PPP compared to Black PPP [10], it is likely that many of the cases are undetected due to biases in screening, identification, referral, and access to PMAD and PSUD treatment among Black PPP [11]. Indeed, Black PPP are less likely to be screened and attend treatment for PMADs and PSUDs compared to White PPP [12–16], resulting in significant under-recognition of PMADs and PSUDs and thus greater untreated illness among Black birthing people. National survey data demonstrate that rates of past month substance use among Black women are higher than national averages [11, 17], but Black PPP are less likely to receive substance use treatment than White PPP [18]. Black birthing people are also more likely to experience delays in care and are less likely to receive follow-up treatment or fill prescriptions for postpartum mental health concerns than their White counterparts [16, 19], despite having higher incidence of hospitalizations for postpartum depression [20]. Black PPP are also less likely to receive medications for opioid use disorder (MOUD), and when they receive MOUD, may be undertreated compared to White PPP [21]. Further, a significant contributor to racial/ethnic disparities in MOUD receipt is delayed recognition and diagnosis of PSUD, with diagnosis occurring 37 days later in racially/ethnically minoritized pregnant people relative to White pregnant people [22]. Evidently, this robust body of nationally representative and empirical literature supports the assertion that there are significant disparities in screening, identification, and treatment referral of PMADs and PSUDs for Black birthing people.

In addition to low rates of screening and referral, all pregnant people who use drugs face significant barriers to disclosing prenatal substance use due to punitive policies involving the criminal legal and child welfare systems. In the U.S., prenatal drug use is considered a crime punishable by law in three states, and 18 states consider prenatal substance exposure to be child abuse. Punitive policies that conflate substance use in pregnancy with child abuse/neglect and criminalize addiction have unintended consequences including further stigmatization and marginalization, and therefore are significant barriers to prenatal substance use disclosure. Substance use along with mental health problems and intimate partner violence are the leading reasons for reports to child welfare authorities [23], highlighting the complex and multifaceted problems that birthing people involved with the child welfare system face. Black birthing people are overrepresented within the child welfare system across all 50 states [24], and consequently, Black children are disproportionately involved in the foster care system [25]. System and provider level factors account for these disparities. Despite overall lower rates of PSUD screening, toxicology testing (e.g., urine drug screening) is frequently applied unevenly across races due to lack of standardized testing procedures and provider bias, such that pregnant persons of color are drug tested in situations where White pregnant people are not. To demonstrate, Black pregnant people experience selective drug screening at a rate greater than 4 times that of White pregnant people (despite being significantly less likely to test positive for drugs) [26]. Targeted toxicology testing results in a greater proportion of Black birthing persons being reported to child welfare authorities and experiencing criminal legal repercussions of prenatal substance use, including incarceration and child separation immediately postpartum [27]. Black birthing people are also more likely to suffer loss of custody and less likely to be reunited with their children than White birthing people involved in the child welfare system [27]. Universal toxicology screening during birthing hospitalizations eliminates racial disparities in drug screening [26], creating a more equitable system wherein Black birthing people do not disproportionately experience loss of custody and criminalization. Further, universal PSUD screening reduces racial disparities by ensuring equitable screening across race and ethnicity and allowing for a greater proportion of Black pregnant people to receive PSUD treatment, thereby reducing child welfare involvement at delivery [26, 28].

Homicide is another leading cause of maternal mortality, exceeding the leading obstetric causes of maternal mortality (e.g., hypertensive disorders of pregnancy) more than twofold [29]. Many pregnancy-associated homicides are perpetrated by a current or previous romantic partner, and thus occur in the context of intimate partner violence (IPV) [30, 31]. IPV includes physical or sexual violence, stalking, and psychological aggression and coercion (including reproductive coercion) by a current or former intimate partner [32], and is linked to myriad chronic physical (e.g., diabetes, chronic pain) and mental health (e.g., posttraumatic stress, substance use) conditions [33, 34]. The estimated rate of pregnancy-associated homicide ranges from 2.9 to 6.2 per 100,000 [35], and IPV experiences in pregnancy vary widely from 3.2 to 28% depending on sociodemographic factors, screening measures, and definitions [36]. Perhaps counterintuitively, pregnancy is not protective against IPV experiences. There is a significantly higher risk for homicide in pregnancy and the first postpartum year compared with females of reproductive age who are neither pregnant nor postpartum [29]. A recent analysis of over 4,700 mortality files from the National Center for Health Statistics suggests that the elevated risk for homicide during pregnancy was present only in non-Hispanic Black pregnant people, while individuals with other racial and ethnic identities did not experience this increased risk [29]. Studies published over the last 30 years consistently find that risk for homicide in the peripartum period is most pronounced among Black individuals, with a staggering 5- to 11-fold increase in risk (depending on intersecting demographic factors such as age and relationship status) relative to White peripartum people [29, 37, 38]. Additionally, the National Center for Health Statistics reports that across all individuals of reproductive age who died by homicide, peripartum decedents were more likely to be Black [29].

These vast disparities in rates of and risk for pregnancy-associated homicide and IPV are in part due to lack of routine IPV screening and structural inequities and biases embedded within obstetric and other healthcare settings. Increased access to firearms is strongly associated with intimate partner homicide and most IPV deaths involve firearms [31]; therefore policies around controlling access to firearms in circumstances of gender-based and domestic violence will also likely decrease rates of IPV, in addition to universal screening [39, 40]. For decades, various organizations such as the U.S. Preventative Services Task Force (USPSTF) have recommended universal screening for IPV across medical settings. However, the uptake of these guidelines has widely been poor, largely due to provider factors including discomfort, lack of IPV knowledge, and perceived time constraints [41]. Additionally, racially and ethnically minoritized patients are screened for IPV at significantly lower rates than White patients [42]. Within maternity care settings, systemic and provider biases and discrimination may prevent Black PPP from disclosing IPV experiences and accessing the services and support they need [43]. Even when Black PPP are able to access services for IPV, the services are inadequate and ineffective at meeting their needs [44].

Numerous professional organizations, including the American College of Obstetricians and Gynecologists (ACOG) and USPSTF, endorse the need to screen for PMADs, PSUDs, and IPV within routine antenatal and postpartum visits using validated screening tools, and provide referrals to treatment for those who screen positive [45]. For individuals screening positive for substances, a brief intervention utilizing motivational enhancement techniques is employed to encourage behavior change, followed by a referral to substance use treatment when indicated [46, 47]. For positive screens on mental health problems and IPV experiences, relevant resources and treatment referrals are made. Despite its robust evidence base and recommendation by multiple professional organizations, evidence-based screening and treatment referral for PMADs, PSUDs, and IPV is underutilized among obstetric, pediatric, and family medicine practices. Indeed, there are unacceptably low rates of screening for PMADs, PSUDs (15%) and IPV (10–20%) as well as low rates of treatment referral completion (20%) [48–52] among pregnant and postpartum patients. Screening and treatment referral and attendance rates are even lower among Black PPP, as described above.

The behavioral health disparities experienced by Black PPP are driven by the intersection of systemic racism, structural sexism (i.e., systemic gender inequality), and reproductive discrimination [53]. Historical and intergenerational (e.g., medical apartheid), vicarious (e.g., friends and family), and personal experiences (e.g., dismissal of pain, stereotyping) with gendered sexism, reproductive discrimination, and other structural inequities ingrained within healthcare settings have resulted in Black PPP’s profound and legitimate mistrust in providers and the U.S. healthcare system [54, 55]. Racism within healthcare settings contributes to stigmatization by providers, and ultimately, the current reality that Black birthing people are disproportionately involved in the child welfare system, more likely to have parental rights terminated, and drug screened in pregnancy more frequently than their White counterparts [27, 56–59]. Understandably, Black pregnant and postpartum patients experience reluctance to disclose prenatal substance use, IPV, and other stigmatized experiences, which in combination with lower rates of screening by providers [12–16, 52], contributes to less recognition and treatment of PMADs, PSUDs, and IPV among Black individuals. Building upon the effects of anti-Black racism, structural sexism results in more chronic health conditions and worse perceived health among women, but less preventative care seeking [60, 61]. Within reproductive healthcare settings, Black PPP report the absence of shared decision making and experiences of stereotyping, invalidation, and dismissal by professionals, leading to self-preservation acts such as seeking care only when desperate [55]. Consequently, there is great need to produce effective and *equitable* PMAD, PSUD, and IPV screening and referral procedures that can be widely implemented throughout obstetric, pediatric, and family medicine practices.

One promising solution to improve equitable PMAD, PSUD, and IPV screening and treatment referral is through use of innovative technology solutions. Our team previously developed a text/phone-based screening and referral to treatment program for PMADs, PSUDs, and IPV that eliminated racial disparities termed *Listening to Women and Pregnant and Postpartum People (LTWP)*. LTWP employs the same procedures as traditional in-person screening and referral, including screening questions and assessment of PMADs, PSUDs, and IPV, and delivers a brief intervention utilizing motivational interviewing techniques and provides referral resources for IPV and social drivers of health (e.g., childcare needs, food insecurity). LTWP differs from traditional in-person screening and referral in that screening and assessment questions and subsequent feedback are delivered via text to the patient’s phone. Additionally, a care coordinator with a master’s degree in clinical social work provides the brief intervention, completes further assessment, and provides appropriate resources (e.g., housing, food) and treatment referral using shared decision making via phone. In a previously conducted quasi-experimental pilot study of 3,535 PPP who were engaged with prenatal care, LTWP demonstrated a significant reduction in racial disparities compared to in-person screening and referral practices [62]. Specifically, Black PPP were more likely to be screened and screen positive for PMADs and PSUDs with LTWP compared to Black PPP who received in-person screening and referral, suggesting that LTWP may have increased comfort to disclose symptoms. Additionally, Black PPP, compared to White PPP, enrolled in in-person screening and referral were less likely to attend treatment; however, Black and White PPP were equally likely to attend treatment with LTWP [62].

The promising preliminary findings from the LTWP pilot trial suggest that technology enhanced screening and referral practices may reduce racial disparities in PMAD, PSUD, and IPV screening, treatment referral, and engagement in treatment for Black PPP, while still sufficiently addressing the needs of other PPP. A deeper understanding of Black PPP’s experience in the LTWP program that contributed to greater endorsement of mental health and substance use problems is needed to further tailor this prevention and intervention tool. To this end, the purpose of this qualitative study was to identify facilitators of mental health symptom and substance use endorsement and attendance to PMAD and PSUD treatment among Black PPP, in general and with the LTWP program. Gaining the perspectives of Black PPP on their experience with the LTWP tool will contribute to patient-centered, culturally competent, and adaptive maternal mental health and substance use screening and treatment programs that have the potential to reduce the staggering racial disparities in maternal health and morbidity.

## Methods

### Participants and Procedure

The Medical University of South Carolina’s Institutional Review Board (IRB) granted a waiver of written informed consent (Pro # 00085580) and approved all procedures. Participants were recruited nationally through social media public advertising on academic institution pages (Facebook) and TrialFacts, a patient recruitment service that adheres to IRB guidelines. Compensation of a $20 gift card was provided to individuals who referred a potentially eligible participant. Inclusion criteria required participants to self-identify as Black and be currently pregnant or pregnant within the past 24 months. Participants met past or current DSM-5 criteria for any substance use disorder (other than tobacco alone) and/or past or current mental health disorder with peripartum onset, or had no history of a mental health or substance use disorder. People were excluded from the study if they had active psychotic symptoms, were currently experiencing suicidal or homicidal ideation, or were unable to comply with study procedures. In total, 84 participants met criteria for inclusion and enrolled in the study. Sixteen participants were unable to be contacted to schedule and complete the interview and a total of 68 completed the qualitative semi-structured interviews.

Prior to each interview, participants provided verbal consent to be included in the study after a Statement of Research was administered by research staff. Semi-structured interviews were conducted between December 1st 2020 and October 8th 2021 through HIPAA compliant virtual videoconferencing. Two trained, doctoral level, female clinicians who were not previously involved in the participants health care and did not know the study participants conducted the interviews. The interviews were conducted using a comprehensive set of questions developed by the authors that aimed to identify facilitators of mental health symptom endorsement and attendance to PSUD treatment among Black PPP, in general and with the LTWP program (e.g., comfort disclosing mental health and substance use in general and with LTWP, beliefs about importance of mental health screening in pregnancy). Participants did not participate in the LTWP pilot trial and were naïve to the LTWP tool prior to the study. As part of the interview procedures, participants were enrolled in the LTWP program and provided feedback on the tool. Interviews ran for 40 to 60 min (mean duration = 46.5 min), and field notes were taken during sessions to track observations. Participants were compensated with a $20 gift card following the completion of the interview. An additional $20 gift card was given to participants who attended the first scheduled interview and did not ‘no show’ or cancel the interview within the prior 24 h.

### Data Analysis

Participant interviews were audio recorded and transcribed verbatim. All participant data was stored securely in REDCap and NVivo 12 software was used for data management and analysis. The Consolidated Criteria for Reporting Qualitative Research (COREQ) guided reporting of methods and results [63].

The grounded theory approach was used to conduct a qualitative content analysis [64, 65], identifying and classifying themes that naturally emerged from the data of participant perspectives [66]. A three-step inductive approach was used to form a comprehensive codebook. (1) Responses were carefully examined to capture all possible emerging themes. (2) A codebook was used by two independent coders to code and analyze participant’s responses to interview questions [65, 67]; coders were able to apply more than one code if applicable. Overall interrater reliability was 95% (range of 91–99%) and any discrepancies were discussed and resolved by the two coders. (3) Themes that emerged were refined and merged to create a comprehensive codebook.

## Results

Participants had an average age of 31.2 years (*SD* = 4.3), all self-identified as Black (100%), and a minority reported Hispanic identity (3.5%). Most reported being married, engaged, or living with their partner as a couple (58%), and many had completed college (42.4%). Overall, 42.6% of participants reported having a PMAD, 36.8% a PSUD, and 20.6% reported no psychiatric history.

Four overarching themes regarding the LTWP tool, each with their own sub-themes, emerged from the participants’ answers to the semi-structured interview questions including (1) Usability of the tool; (2) Comfort with the tool; (3) Necessity of the tool; and (4) Recommendations for the tool. Each is described below, with the proportion of interview time spent on each theme and sub-theme depicted in Table 1, and representative quotes displayed in Table 2.

**Table 1:** Percent of time discussed for each theme and sub-theme

Theme	Percentage of Time Discussed
Usability of the tool Navigation Clarity of information Ease of use Length of the tool Service and data availability Comfort with the tool Comfort discussing mental health using the tool Comfort discussing substance use using the tool Confidentiality and privacy Care coordinator Necessity of the tool Necessary to screen/access Necessary to remove stigma Recommendations for the tool Revise wording of some questions Add information about confidentiality	53.7% 7.1% 4.8% 14.8% 4.8% 9.5% 42.1% 11.0% 10.1% 8.3% 12.8% 24.5% 20.8% 2.8% 4.2% 1.4% 2.7%

**Table 2:** Representative quotes for each theme and sub-theme for the listening to women and pregnant and postpartum people tool

Code	Reflective quotes
**Theme 1: Usability of the mobile-based tool**	
Navigation	*“Once I got the text message, I just clicked the little blue link and it took me straight there. That was easy.”*
Clarity of information	*“I think it’s straightforward enough to not get it confused…”* *“I thought it was super direct in the descriptions about the drugs…That’s helpful too.”*
Ease of use/user friendly	*“Yes, that was the best part about it. I could sit here with my phone in my hand and not have to do a whole lot. Just click and go, click and go.”* *“The technology part of it seemed to be pretty easy, pretty user friendly.”*
Length of tool	*“It’s not too long. It’s appropriate.”*
Service and data availability	*“I don’t have any storage to do that.”* *“I have internet where I live. I drive a dump truck, so there’s no internet in my truck.”*
**Theme 2: Comfort using the mobile-based tool**	
Comfort discussing mental health using tool	*“I did feel comfortable. I always feel like it’s easier to answer them over the phone instead of face-to-face.”*
Comfort discussing substance use using tool	*“The substance abuse questions were fine…but to be open about something like that, it’s an uncomfortable subject.”*
Confidentiality/Privacy	*“I can express myself privately because you’re not seeing me. I don’t have to be scared of something.”*
Care Coordinator	*“Yes, definitely. Because I wouldn’t have to search to try to find some place to get help. I think that’s one of the major problems… because at the hospital that I work for you can’t come in and say, “I want to come in. I want to sign myself up for a detox or substance use or mental health.” You actually have to be referred or you have to be admitted through the ER … and you have to meet the criteria in order to be admitted as an inpatient.”*
**Theme 3: Necessity of the mobile-based tool**	
Necessary to screen/access	*“I think it’s … getting a comfortable environment for people to truly be honest about their mental health or substance abuse… it’s providing a clear and quick way to address it. Instead of having to come in and set up an appointment, a message could just go out. Especially with being a mom with a newborn, you could take the time and do the survey and really answer those questions if need be, compared to trying to come in and schedule an appointment…”*
Necessary to remove stigma	“It’s so important because a lot of times as women we always have to be the strong one. Sometimes we don’t speak up because we’re feeling weak or… we can’t do the task at hand, so it makes us feel guilty, which I definitely felt. And it makes you feel sometimes that people are going to view you differently.” “I definitely think it’s something that we, as a Black community, should work on because a lot of people suffer from mental health, a lot of people.” “I think we should talk about how mental health is there these days. Because some people they’re really suffering in silence…” “Being able to break the mental health stigma and have people know that they’re not the only ones that are going through something. It’s just not greatly talked about in our community and shared amongst each other. If you address the issue right away, as soon as you can, it doesn’t get as bad as it could get.”
**Theme 4: Recommendations for the mobile-based tool**	
Revise wording	*“I would probably put the questions about your family a little bit further down the list…”* *“One of the questions says this past month, how many days per month did you drink, and then on the next question, it says, how many drinks on any given day.”*
Add information about confidentiality	*“If the question is very sensitive and you’re not assured of how your information would be [protected], that tends to make you insecure. But if you were assured that the information that you give out will be kept confidential, it won’t be exposed to even your friends or relatives, that’s going to be comfortable.”*

### Theme 1: Usability of The Mobile-Based Tool

All participants discussed usability of the mobile-based tool throughout the semi-structured interviews, which constituted approximately half of the duration of interviews. Sub-themes related to usability include comments on navigation, clarity of information, ease of use, length of the tool, and service and data availability. Specifically, participants mentioned that the mobile-based tool was easy to navigate, and all participants reported the information within the mobile-based tool was extremely clear and the text messages were easy to understand. The majority of participants made statements that the mobile-based tool was user friendly, ‘quick, convenient and easy to understand’, and appreciated the brief length of the tool. Participants remarked that they liked that they could use the tool from the privacy and comfort of their own homes. However, a few participants (< 10%) stated that they did not have enough data to use the tool, or that they did not always have access to cell service or Wi-Fi to complete the questions. Over 90% of participants had the requisite access to technology to use the tool as it was intended.

### Theme 2: Comfort Using The Mobile-Based Tool

All participants reported that they were comfortable utilizing the mobile-based tool to discuss their mental health and substance use. Participants shared the sentiment that the text message interface increased their level of comfort in disclosing mental health and substance use relative to face-to-face with a provider, as the tool removed fear from judgement and other social consequences typically experienced when discussing PMADs and PSUDs with providers. Additionally, answering questions over text was perceived as more private and confidential than in-person. However, participants emphasized the importance of explaining confidentiality prior to answering the questions to increase perception of the confidentiality of their responses. Finally, most participants reported they would be comfortable and even preferred contacting or being contacted by the care coordinator and that this would create a more efficient and effective way to get care. Participants explained that having the care coordinator facilitate their referral to social or behavioral health services would remove the burden of searching for a provider, in addition to eliminating other barriers (e.g., needing a referral for specialty services, lack of knowledge about or access to referral information).

### Theme 3: Necessity of The Mobile-Based Tool

The majority of participants revealed that the mobile-based tool would be beneficial in helping to screen and assess PMADs and PSUDs, and particularly within the Black community. Participants described that having a newborn prevented them from easily scheduling or attending appointments, but the text messages removed this barrier and allowed them to still receive the help they needed. The LTWP tool was described as providing a comfortable and confidential environment for Black PPP to disclose their mental health and/or substance use, learn that these experiences in pregnancy and postpartum are common, and to have postpartum mental health difficulties and their disclosure normalized and validated. The tool was seen as helpful for disseminating information related to PMADs and PSUDs, and for potentially helping to educate communities on these conditions and provide referrals to resources and treatment that are accessible and available to patients. Many participants specifically reported that Black communities could benefit from this type of tool to help lessen the “stigma” of mental illness and provide easily accessible resources and referrals to these populations.

### Theme 4: Recommendations for The Mobile-Based Tool

Only a few participants discussed recommendations for the mobile-based tool, which included rewording some of the questions and adding information to the program about confidentiality. Some participants suggested rephrasing the wording of certain questions to be clearer or more consistent (e.g., improve consistency in reporting of numbers of alcoholic drinks per day/week), as well as moving family history questions on the 4Ps scale—a widely used screening instrument for substance use in pregnancy [68]—to the end so as not to “turn off” patients by initially asking about personal family history without any prior context. A small minority of participants indicated they preferred greater or more direct assurances that the information provided within the LTWP tool is kept confidential, which would help patients feel more comfortable answering sensitive questions.

## Discussion

Qualitative interviews with Black PPP were conducted to gain a deeper understanding of how the LTWP text/mobile-based screening and referral program for perinatal mental health and substance use disorders may have reduced racial disparities, as evidenced in a previous quasi-experimental trial [62]. Complementing the pilot trial findings, our qualitative results showcase the importance of culturally tailored screening and referral programs for PMADs and PSUDs. Specifically, Black PPP identified that there is a strong need for a tool like LTWP in their communities as it provides much needed information to normalize and de-stigmatize the experience of PMADs and PSUDs. Black PPP liked the ease of use and brevity of the text-based screening and that they could complete it from their own home, which instilled an elevated sense of privacy and confidentiality relative to in-person services. Finally, participants preferred the care coordinator facilitate their behavioral health services, describing that this reduced barriers to receiving care. Altogether, feedback from our participants demonstrates how the LTWP tool, and possibly other technology-adapted screening and referral programs, may reduce barriers such as limited access and stigma, and create a context where Black PPP are comfortable sharing information about their mental and behavioral health.

Racial discrimination is common among African Americans (e.g., reported by approximately three-in-four Black persons in the U.S. [69]), including in the form of discriminatory acts, microaggressions, and racial slurs, and in the context of police interactions, employment, and healthcare settings, among others [70]. Black women and birthing people face even greater discrimination due to their intersectional identities, resulting in devastating consequences including heightened Black maternal and infant morbidity and mortality [71–73]. Anti-Black racism, sexism, and reproductive discrimination faced by Black PPP result in stigmatization of Black motherhood and devaluation of Black pregnancies. Further, Black peripartum patients are subjected to racist stereotypes and assumptions by their health care providers, which reduces efficacy of and access to quality services while simultaneously causing significant stress [74]. Racial discrimination is one of the most common contributing factors to pregnancy-related deaths in the U.S. [54, 55, 75], and is deeply intertwined with discrimination on the basis of perinatal substance use and mental health disorders [59, 76]. In the state of South Carolina where the LTWP study took place, discrimination is the most common contributing factor to pregnancy-related deaths, occurring in 55.6% of deaths [77]. Not distinct from rates of discrimination, substance use disorders and mental health conditions are also prevalent among South Carolina pregnancy-related deaths (28.9% and 25.0%, respectively) [77], highlighting the cumulative impact of intersecting gendered racism and behavioral health discrimination on maternal morbidity and mortality [78].

Discrimination and other forms of racism manifest in disparities in care, clinical communication, and shared decision-making, significantly impacting the quality of and access to health care for Black PPP [79, 80]. Discrimination in healthcare settings leads Black PPP to experience greater reluctance to ask questions, communicate openly, accept care during labor/delivery, and attend postnatal appointments [81, 82]. Black and other women of color with IPV experiences report that racism, stigma, fear of judgment, and not expecting assistance from providers due to experiences of racism, prevent them from disclosing IPV [83]. In addition, substance use and mental health conditions, particularly in pregnancy, are among the most stigmatized of all health conditions, with Black PPP experiencing even greater stigma than their White counterparts [84–86]. LTWP appears to help diminish some of these barriers. Given the confidential and de-stigmatized text-message screening interface, Black PPP who enrolled in the LTWP program described fewer barriers to open communication and an increased sense of comfort and confidentiality allowing them to engage with treatment referrals, which they are *449% less likely* to do with face-to-face screening and referral, compared to LTWP [62]. These qualitative findings illuminate how technology-based adaptations to behavioral health screening and referral can reduce perceived negative judgment and facilitate symptom identification and referral to treatment, thereby more adequately meeting needs of Black PPP.

Black PPP also identified that the LTWP program could help reduce social stigma within their communities, where mental health and substance use are frequently underrecognized, misunderstood, not discussed, and seen as “taboo.” Several participants indicated that despite mental health concerns being prevalent within their communities, the considerable stigma, especially for Black mothers and birthing people, causes people to “suffer in silence.” In addition to stigma serving as a barrier to treatment seeking, it also prevents individuals from seeking or obtaining support from their families, significant others, or other social supports [87, 88]. Qualitative research suggests that individuals from Black communities are reluctant to seek treatment for substance use disorders for fear of being judged and labeled as a criminal or addict by their family and peers, and due to embarrassment and shame [89]. Others perceive mental health and substance use as a private matter that should stay within the family, and do not trust the healthcare system given the intergenerational discrimination, bias, and racism experienced by Black individuals at the hands of U.S. healthcare system. However, limited access to accurate information about and services for mental health and substance use further contribute to social stigma within Black communities [90]. LTWP provides a simple and cost-effective way to disseminate information and resources for PMADs, PSUDs, and IPV to members of the Black community (as well as other minoritized communities and White individuals). Additionally, through screening for PMADs and PSUDs, LTWP normalizes the experience of mental health concerns in the peripartum period and imbues a sense of not being alone in these experiences, which participants expressed would be beneficial to them individually and to their communities as a whole.

This study provides novel insight into an innovative solution to improve Black PPP’s comfort with mental health symptom disclosure and access to PMAD and PSUD treatment; however, there are several limitations that may limit generalizability of participants’ experience with the LTWP tool. First, the sample may primarily represent college educated Black/African American birthing people, with 42% having completed college. Given the socioeconomic conditions for attending college (e.g., paying tuition, family history of college education) and those that follow college completion (e.g., higher income), this sample may not represent the lived experience of all Black birthing people with PMADs, PSUDs, or experiences with IPV. Second, the study was conducted with a sample of PPP individuals who were recruited nationally. Variability in child welfare reporting related to prenatal substance use and other maternal health conditions may have impacted participant experiences, which may not generalize to all U.S. states. Further, given the diversity of Black communities throughout the U.S., it is likely that not all viewpoints are represented in these data. It will be important for the implementation of LTWP (and other related programs) to allow for local adjustments to account for nuances in the many diverse Black communities across the U.S. Third, by nature of the internet-based recruitment and inclusion criteria, the study was biased toward individuals with access to technology and those who speak English, respectively. Individuals who have consistent access to technology are inherently different from those who do not [91, 92], and individuals with an existing interest in mental health may have been more likely to participate. It is important that future research incorporate other marginalized, minoritized, and non-English speaking populations to assess for additional modifications needed for generalizability. Finally, although the double coding technique [65] was used to ensure reliability while conducting the qualitative analysis, unique perceptions and unconscious biases may have influenced data extracted by the coders.

Technology-enhanced screening and intervention for PMADs, PSUDs, IPV, and other maternal health factors within obstetric settings are being piloted across the U.S. Pilot work has indicated that electronic screening and treatment referral is feasible in prenatal clinic settings, acceptable to pregnant and postpartum patients [93], and a cost-effective alternative to in-person screening and referral [94]. A patient-facing, tablet-based screening tool, *SmartStart*, assesses maternal health risk (e.g., behavioral health, diabetes) and protective factors (e.g., vaccinations) and was preliminarily integrated within obstetric settings [95]. A computer-based screening and referral tool for PSUDs that utilizes a three-dimensional cartoon character “guide” was equally effective in reducing days of substance use as in-person screening and referral, and both screening/referral groups were more effective than an informational handout with referrals [51]. Finally, patients in a prenatal clinic receiving both computer-based and provider administered IPV screening indicated the computer screening allowed for disclosure without fear of judgment while the provider screening provided an avenue for building an emotional connection [96]. Our team is the first to find that technology-facilitated screening and referral can improve the disparities in PMAD and PSUD screening, treatment referral, and engagement in behavioral health services [62]. LTWP has the possibility to empower Black PPP, heal their communities, and achieve health equity. Current studies are underway to further evaluate the potential of LTWP in reducing disparities in maternal health, including a randomized controlled trial funded by the Patient-Centered Outcomes Research Institute to evaluate whether LTWP can reduce the need for emergency department visits within the first postpartum year for postpartum complications among South Carolina Medicaid beneficiaries (*Leveraging Technology to Reduce Disparities and Improve Early Detection of and Timely Care for Postpartum Complications*). Together, this nascent, yet growing body of literature highlights the value of technology-enhanced screening and referral programs for both PPP and providers and is an important area of continued research.

## Conclusion

The maternal morbidity and mortality rate in the United States is the highest among developed countries, and Black women and birthing people are four times more likely to die during pregnancy, childbirth, or the first year postpartum than their White counterparts [1, 2]. Policy changes (e.g., universal pre-conception healthcare; decriminalization of prenatal substance use and emphasizing treatment, not punitive action) and cultural and institutional changes that reduce, bias, discrimination, and individual, institutional, and structural gendered racism are needed to ensure equality and equity for all birthing people. One small but promising step toward improving maternal behavioral health at the provider/practice-level is implementing technology-facilitated screening and referral to treatment for PMADs, PSUDs, and IPV within obstetric, pediatric, and primary care offices. In this qualitative study, Black PPP indicated that mobile/text-based screening, brief intervention, and referral to treatment was acceptable, user-friendly, convenient, and confidential, which helped them feel comfortable to disclose mental health and/or substance use. This type of innovative resource is likely to facilitate engagement in follow-up care, as evidenced by previous work [62]. Future work leveraging implementation science is needed to integrate and widely disseminate LTWP and other technology-based screening and referral programs into routine pre- and postnatal practice. Doing so has the potential to reduce racial disparities in PMAD, PSUD, and IPV screening and treatment referral and acceptance, thus reducing Black maternal mortality in the United States.
